# Which behaviour change techniques are effective to promote physical activity and reduce sedentary behaviour in adults: a factorial randomized trial of an e- and m-health intervention

**DOI:** 10.1186/s12966-020-01001-x

**Published:** 2020-10-07

**Authors:** Helene Schroé, Delfien Van Dyck, Annick De Paepe, Louise Poppe, Wen Wei Loh, Maïté Verloigne, Tom Loeys, Ilse De Bourdeaudhuij, Geert Crombez

**Affiliations:** 1 Ghent Health Psychology Lab, Department of Experimental-Clinical and Health Psychology, Faculty of Psychology and Educational Sciences, Ghent University, Henri Dunantlaan 2, Ghent, 9000 Belgium; 2 Research Group Physical Activity and Health, Department of Movement and Sports Sciences, Faculty of Medicine and Health, Ghent University, Watersportlaan 2, 9000 Ghent, Belgium; 3 Department of Public Health and Primary Care, Faculty of Medicine and Health Sciences, Ghent University, Ghent, Belgium; 4 Department of Data Analysis, Faculty of Psychology and Educational Sciences, Ghent University, Ghent, Belgium

**Keywords:** E-health, M-health, Self-regulation, Behaviour change techniques, Physical activity, Sedentary behaviour, Factorial trial

## Abstract

**Background:**

E- and m-health interventions are promising to change health behaviour. Many of these interventions use a large variety of behaviour change techniques (BCTs), but it’s not known which BCTs or which combination of BCTs contribute to their efficacy. Therefore, this experimental study investigated the efficacy of three BCTs (i.e. action planning, coping planning and self-monitoring) and their combinations on physical activity (PA) and sedentary behaviour (SB) against a background set of other BCTs.

**Methods:**

In a 2 (action planning: present vs absent) × 2 (coping planning: present vs absent) × 2 (self-monitoring: present vs absent) factorial trial, 473 adults from the general population used the self-regulation based e- and m-health intervention ‘MyPlan2.0’ for five weeks. All combinations of BCTs were considered, resulting in eight groups. Participants selected their preferred target behaviour, either PA (*n* = 335, age = 35.8, 28.1% men) or SB (*n* = 138, age = 37.8, 37.7% men), and were then randomly allocated to the experimental groups. Levels of PA (MVPA in minutes/week) or SB (total sedentary time in hours/day) were assessed at baseline and post-intervention using self-reported questionnaires. Linear mixed-effect models were fitted to assess the impact of the different combinations of the BCTs on PA and SB.

**Results:**

First, *overall efficacy* of each BCT was examined. The delivery of self-monitoring increased PA (t = 2.735, *p* = 0.007) and reduced SB (t = − 2.573, *p* = 0.012) compared with no delivery of self-monitoring. Also, the delivery of coping planning increased PA (t = 2.302, *p* = 0.022) compared with no delivery of coping planning. Second, we investigated to what extent *adding BCTs increased efficacy*. Using the combination of the three BCTs was most effective to increase PA (x^2^ = 8849, *p* = 0.003) whereas the combination of action planning and self-monitoring was most effective to decrease SB (x^2^ = 3.918, *p* = 0.048). To increase PA, action planning was always more effective in combination with coping planning (x^2^ = 5.590, *p* = 0.014; x^2^ = 17.722, *p* < 0.001; x^2^ = 4.552, *p* = 0.033) compared with using action planning without coping planning. Of note, the use of action planning alone reduced PA compared with using coping planning alone (x^2^ = 4.389, *p* = 0.031) and self-monitoring alone (x^2^ = 8.858, *p* = 003), respectively.

**Conclusions:**

This study provides indications that different (combinations of) BCTs may be effective to promote PA and reduce SB. More experimental research to investigate the effectiveness of BCTs is needed, which can contribute to improved design and more effective e- and m-health interventions in the future.

**Trial registration:**

This study was preregistered as a clinical trial (ID number: NCT03274271). Release date: 20 October 2017.

## Background

The prevalence of non-communicable diseases such as type 2 diabetes, cancer, osteoarthritis, depression and cardiovascular diseases in adults is high and rising [1]. These diseases are often attributed to unhealthy lifestyles [1]. Increasing physical activity (PA) and reducing sedentary behaviour (SB) are key to reduce the burden of these diseases [2–5]. The recommendation of the World Health Organisation for PA is to engage in at least 150 min PA at moderate to vigorous intensity each week [6, 7]. However, worldwide 31.1% of adults is insufficiently physically active [8]. For SB, only a few countries have formulated recommendations. For example, in Belgium, adults are recommended to restrict their sitting time to a maximum of 8 h per day and to interrupt sitting every 20 to 30 min [6]. Notwithstanding, Belgian adults sit on average 8.3 h a day [9]. This is in line with other studies revealing that adults spend on average 8.5 h sitting a day [10]. As such, there is a clear need to develop effective interventions that promote PA and reduce SB on a large scale.

The use of technologies, such as electronic (e-) or mobile (m-) health interventions, is one strategy to promote health behaviour. E- and m- health is defined as “the use of information and communications technology, especially the internet, to improve or enable health and health care” [11]. These interventions are promising: They can use a personal and interactive approach while reaching a large group of participants at a relatively low cost [12, 13].

Commercial and theory-based e- and m-health interventions are available. Most of them target only the intention towards health behaviour (e.g. by targeting knowledge) [14]. However, intentions do not always translate into the actual behaviour [15], a phenomenon known as the “intention-behaviour gap” [16]. Several theoretical models have proposed ways to bridge this gap. These models, amongst which self-regulation models [16, 17], guide individuals to change their behaviour, beginning with the development of an intention over the actual adoption of the behaviour to finally behaviour maintenance [18]. In doing so, self-regulation models, as for example the Health Action Process Approach (HAPA) model [19], address not only pre-intentional but also post-intentional processes of behaviour change.

Several behaviour change techniques (BCTs) may target post-intentional processes. In this paper, the focus is on ‘action planning’, ‘coping planning’ and ‘self-monitoring’ [19, 20]. These techniques are key within the HAPA-model and are well established to bridge the intention-behaviour gap [15, 19, 21, 22]. The HAPA model is frequently used for the promotion of PA [15, 21, 23] in both clinical [24, 25] and non-clinical populations. The model is generic and flexible, and can easily be applied to several behaviours [26] such as dietary behaviour [19, 26], sunscreen use [27], *and sedentary behaviour* [28–30]. Therefore we chose to focus on the BCTs ‘action planning’, ‘coping planning’ and ‘self-monitoring’ for both PA and SB.

BCTs are most often used as part of complex interventions, consisting of many other techniques. The efficacy of single BCTs or particular combinations of BCTs remains unclear. There are some indications in the literature that some BCTs are better than others. In their meta-analysis, Michie et al. [31] reported that ‘self-monitoring’ combined with at least one other technique was more effective in changing healthy eating and physical activity than interventions that did not include ‘self-monitoring’. Nonetheless, firm conclusions are not yet possible, because of the non-experimental nature of the design, precluding cause-effect inferences. What is needed is *experimental research* investigating the efficacy of single BCTs, or the efficacy of combinations of BCTs. Such studies are largely lacking [31, 32]. Furthermore, insight into the efficacy of single BCTs can provide guidance in developing future interventions. Only including effective BCTs may create more efficient interventions [33]. This is important for e- and m-health interventions as they often suffer from high attrition rates (60–80%) [34]. Interventions with many BTCs may make interventions time-consuming, less engaging and more confusing [35, 36]. Including only effective techniques may be promising to lower the burden on participants and to reduce attrition rates.

Therefore, the aim of this study was to investigate the specific role of three BCTs that target post-intentional processes [19] in changing PA and SB. In a first step, we investigated the overall efficacy of each of the three BCTs (i.e. action planning, coping planning, self-monitoring) in an e- and m-health intervention. In a second step, we used an additive approach to identify the effective BCTs or their combinations. We investigated to what extent adding BCTs increased efficacy. We applied an experimental approach and conducted a factorial trial using the e- and m-health intervention to increase PA and reduce SB among adults.

## Methods

### Study design

A 2x2x2 factorial randomized controlled trial was carried out to evaluate the efficacy of three BCTs (action planning, coping planning and self-monitoring) and their combinations [32]. The protocol was preregistered [37]. Participants were randomly allocated to one of eight experimental groups, with each experimental group receiving a unique combination of either none, one, two or all three BCTs (see Table 1). The CONSORT 2010 checklist and TIDieR checklist were taken into account [38, 39]; the completed checklists are included as Additional files 1 and 2.

**Table 1:** Combinations of the three BCTs for each experimental group

	Action planning	Coping planning	Self-monitoring
Group 1	+	+	+
Group 2	+	+	–
Group 3	+	–	+
Group 4	–	+	+
Group 5	+	–	–
Group 6	–	+	–
Group 7	–	–	+
Group 8 (control group)	–	–	–

A plus (“+”) symbol indicates that the experimental group received that BCT while a minus (“-“) symbol denotes that the experimental group did not receive that technique. All eight possible combinations of the three BCTs were considered

### Participants and procedure

Adults (18–70 year) were recruited either in person by researchers at the city library of Ghent (which is also a meeting place and strongly focusses on innovation and research in collaboration with Ghent University), or through social media (i.e. Facebook) between February 2018 and December 2018. Only Dutch-speaking adults who had internet access at home and/or work and owned a smartphone (iOS or Android) were eligible. In addition, individuals had to complete the ‘Physical Activity Readiness Questionnaire’ (PAR-Q) [40] as a screening instrument to detect individuals at risk for adverse effects when being more physically active (e.g. individuals with heart problems). The PAR-Q is a self-report instrument consisting of seven items with ‘yes’ or ‘no’ answers. Only those who answered ‘no’ to all items were eligible for the study. Eligible participants were then provided with more information about the study either in person (for those recruited at the library) or via email (for those recruited via social media).

**Fig. 1:**
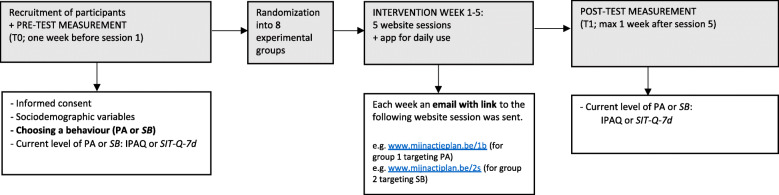
Procedure of the study

The intervention lasted for 5 weeks. It consisted of 5 weekly website sessions and a mobile application, which could be used at any time during these 5 weeks. The procedure of the study is shown in Fig. 1. Participants were measured at 2 time points; pre-test (T0) and post-test (T1). The pre-test measurements were conducted via an online survey tool (Limesurvey, 2017), and included: 1) an agreement requiring them to provide informed consent, 2) sociodemographic information, 3) information about the difference between PA and SB, followed by the question about whether they wanted to improve PA or SB, and 4) questions assessing their current PA or SB level respectively (depending on their response to the previous question). Subsequently, participants were instructed on how to download the “MyPlan” (i.e. the English translation of “Mijn Actieplan”) app on the iOS AppStore or the Android PlayStore. Thereafter, participants were randomly assigned by a researcher (HS) to one of the eight experimental groups using block randomization (on the website www.randomization.com) [37]. The study was single-blinded: participants did not know which experimental group they were allocated to, but the researcher did [37]. After allocation, participants received an email with a link to the intervention and further instructions.

One week after each intervention session, an automatic email to encourage people to start with the following weekly session was sent. Participants who did not complete a session, were sent a reminder. If they had not completed a session after two weeks, they were phoned by the researcher (HS). When there was no reaction after 3 weeks, the participant was considered as a drop-out. Approximately one week after completing all 5 sessions, an email for post-test measurements (T1) was sent to the participants. Post-test measurements included the same questions to assess their PA or SB level (depending on what they had chosen at the pre-test measurement).

As stated in the protocol [37], a power analysis indicated a sample size of 260 participants, assuming an effect size of 0.19 [41], an alpha of 0.05 and a power of 0.80. The required sample size was calculated using GPower 3.1.9.2 [42]. Taken attrition into account, we aimed to recruit approximately 480 participants [37]. The factorial trial was approved by the Committee of Medical Ethics of the Ghent University Hospital (ID number: NCT03274271).

### Intervention MyPlan 2.0

MyPlan 2.0 is an e-and m-health intervention consisting of a website and a mobile application to increase PA and reduce SB. MyPlan 2.0 is based on a previous e-health intervention MyPlan 1.0 which was found to be effective for improving health behaviour [43, 44], but had high attrition rates (78.2%) [45]. Therefore, in MyPlan 2.0 a number of changes were implemented based upon users’ needs and feedback [45, 46]. MyPlan 2.0 is based on the Health Action Process Approach model [19], and includes BCTs such as goal setting, providing information on consequences of behaviour, providing feedback on performance, social support, action planning, coping planning, self-monitoring and reviewing behaviour goals. The BCTs are defined according to Michie et al. [20], Schwarzer et al. [47], Sniehotta et al. [48] and can be found in Table 2. In this study, only the effects of action planning, coping planning and self-monitoring were experimentally investigated (Table 1) because they are well known to address post-intentional processes by bridging the intention-behaviour gap [19]. Before a participant started with the intervention, they already chose at pre-test measurement which of the two behaviours (PA or SB) they wanted to improve (i.e. *goal setting*). This was done to create a feeling of ‘goal-ownership’ [20, 49]. Depending on the chosen behaviour, the participant was directed to MyPlan 2.0 either targeting PA or SB. The two versions of the programme have an identical structure and offer the same BCTs, only the content was adapted to either PA or SB. Additional files 3 and 4 provide screenshots of the website and the mobile app.

**Table 2:** The definitions of the behaviour change techniques used in MyPlan 2.0

Behaviour change technique (label as described in MyPlan 2.0)	Behaviour change technique (label according to the reference ^a, b or c^)	Definition
Goal setting	Goal setting (behaviour) ^a^	The person is encouraged to make a behavioural resolution (e.g. take more exercise next week). This is directed towards encouraging people to decide to change or maintain change.
Providing information on consequences of behaviour	Provide information on consequences of behaviour^a^	Information about the relationship between the behaviour and its possible or likely consequences in the general case, usually based on epidemiological data, and not personalised for the individual.
Providing feedback on performance	Provide feedback on performance^a^	This involves providing the participant with data about their own recorded behaviour or commenting on a person’s behavioural performance (e.g. identifying a discrepancy with between behavioural performance and a set goal) or a discrepancy between one’s own performance in relation to others’.
Social support	Plan social support/social change^a^	Involves prompting the person to plan how to elicit social support from other people to help him/her achieve their target behaviour/outcome.
Action planning	Action planning^b^	Action planning specifies in detail how and under what situational circumstances an intended action is to be implemented. An action plan usually consists of concrete ideas about “when,” “where,” and “how” to act for the purpose of the goal intention.
Coping planning	Coping planning^c^	Coping planning can help a person to overcome obstacles and to cope with difficulties by anticipating personal risk situations (i.e. situations that endanger the performance of intended behaviour) and planning coping responses in detail.
Self-monitoring	Prompt self-monitoring *of behaviour*^a^	The person is asked to keep a record of specified behaviour(s) as a method for changing behaviour.
Reviewing behaviour goals	Prompt review of behavioural goals^a^	Involves a review or analysis of the extent to which previously set behavioural goals (e.g. take more exercise next week) were achieved. In most cases, this will follow previous goal setting and an attempt to act on those goals, followed by a revision or readjustment of goals, and/or means to attain them.

Definitions of the BCTs according to Michie et al. 2011^a^ [20], Schwarzer et al. 2003^b^ [47], Sniehotta et al. 2005^c^ [48].

#### The website

In brief, the website consisted of 5 weekly sessions (Fig. 1). In the first session, users had to register with their email-address and create a profile. Every participant received a basic intervention to which the delivery of the BCTs *action planning*, *coping planning* and/or *self-monitoring* was experimentally manipulated (Table 1). The basic intervention consisted of 1) The possibility to fill in a quiz with information about the benefits of the selected target behaviour (increasing PA or decreasing SB) (i.e. *providing information on consequences of behaviour*), 2) Tailored feedback on the current state of their chosen behaviour (i.e. *providing feedback on performance*), 3) The possibility to read about how they could obtain *social support* from their partner, friends, family or colleagues.

With the BCT “*action planning*” participants were prompted to make a plan by specifying *how* they wanted to reach their goal, *what* they wanted do to, *where* and *when* they wanted to do it. For example a set of possible answers to the questions for a participant who chose PA was: 1) “How do you want to be more physically active this week?” and his/her answer could be: “I will be physically active in my leisure time”, 2) “What do you want to do to be more physically active in your leisure time?” Answer: “I will go swimming”, 3)“Where do you want to do it?” Answer: “At the swimming pool 2 blocks from my house”, 4) “When do you want to do it?” Answer: “On Wednesday and Friday evening”.

With the BCT *“coping planning”* participants had to identify difficult situations or barriers they anticipated to experience while changing their behaviour over the course of the intervention. Participants were then given a list of possible solutions for each barrier, from which they could choose a relevant solution for their specific situation. For example a participant who chose PA might have been asked: “What could be a barrier for you this week to be more physically active?” and the answer could be: “It’s very busy at work this week, so I will have no time”. In response a list of possible solutions adjusted to the barrier were offered (e.g. “I will block a moment in my agenda”).

With the BCT *“self-monitoring”* participants were prompted to monitor their behaviour. They were offered several possibilities (e.g. the app, a diary, a calendar, an activity tracker). For example a person who chose PA may have been asked: “How do you want to keep track of how physically active you are this week?” and an answer could be: “Via the app”.

At the end of the first session, the completed plan was summarised in a printable form. After one week, users received an email to start the following session. There were four follow-up sessions (session 2–5) in which all participants, as part of the basic intervention, were required to reflect on their progress of behaviour change of the past week by evaluating their PA or SB goal (i.e. *reviewing behaviour goals*). Furthermore, participants could see their plan from the previous week and were prompted to adapt or maintain their action plan, coping plan and/or self-monitoring method depending on the experimental group they were assigned to.

#### The mobile application

The application was intended to provide support to the participants on a daily basis. It consisted of different modules through which the users could freely navigate. Again, every participant received a basic intervention with 1) a module consisting out of a quiz regarding the benefits of more PA or less SB, 2) a module where they could collect medals for completing website sessions and quizzes. In the other modules, the experimental manipulated BCTs were included as a function of the experimental group they were assigned to.

The BCT “*action planning*” was implemented in the app by providing the users with the option to review and change their plan throughout the week. Furthermore, a message was sent to remind participants about their plan of the day. If a person planned to go swimming on Wednesday and Friday, a message was sent those days as a reminder.

The BCT “*coping planning*” was implemented by providing a list of barriers participants could encounter. When selecting a barrier, a list of specific solutions for this barrier appeared.

The BCT “*self-monitoring*” supported users to monitor their behaviour. At the end of each day, participants received a message where they could rate on a scale from 0 to 5 if they succeeded in their plan of the day. They could also assess an overview of their responses of the week which were displayed in a graph.

A more detailed description of the intervention can be found in the study protocol paper of “MyPlan 2.0” [37]. No changes regarding bug fixes, downtimes, or content changes to the MyPlan 2.0 intervention occurred after trial commencement.

### Measurements

At pre-test (T0), the following **sociodemographic variables** were assessed: age, gender, weight, height, educational level (4 categories), occupation (7 categories) and marital status (4 categories). Weight and height were self-reported.

**Primary outcomes (PA or SB)** were assessed via self-report at pre-test measurement (T0) and post-test measurement (T1). If participants chose to increase their *PA*, the Dutch long version of the ‘International Physical Activity Questionnaire’ (IPAQ) [50] was used to measure total PA and moderate-to-vigorous-intensity PA (MVPA) in four domains (i.e. work, home, leisure time, transport), as stated in the protocol [37]. However in this study, only MVPA in minutes per week (across all domains) is the outcome for PA. MVPA is considered the most important indicator of PA and PA guidelines for adults only address activities of at least moderate to vigorous intensity [7]. The IPAQ has good reliability (ICC range from 0.46 to 0.96) and a fair-to-moderate criterion validity (ρ = 0.30–0.37). As the IPAQ overestimates PA [51], the data were truncated following the method described by Dubuy et al. [52]. If participants chose to reduce their *SB*, the Dutch 7-days sedentary behaviour self-report questionnaire (SIT-Q-7d) [53] was used to measure sedentary time in five domains (meals, transportation, occupation, non-occupational screen time and other sedentary time). Also, here, only sedentary time in hours per day (i.e. the sum of sedentary time spent across all domains) is the outcome for SB, as existing SB guidelines focus on the total time spent sedentary. The SIT-Q-7d shows moderate-to-good test-retest reliability (ICC = 0.77) and moderate-to-good criterion validity (ρ = 0.49) for total sitting time on an average day [54]. The data were truncated at a maximum of 16 h of sedentary time a day [55].

### Statistical analysis

We initially planned to perform an analysis with the data for PA and SB combined by using a standardized PA/SB score (z-score) in order to achieve more power. In that analysis, the selected behaviour (i.e. PA or SB) would have been introduced as a moderator [37]. In later meetings with statistical experts, we decided to perform the analyses separately for PA and SB. This was done for two reasons. First, it was considered less meaningful to interpret the results of the standardized scores. For example, the difference in MVPA between pre- and posttest *in minutes* is more straightforward to interpret (387.42 min MVPA at pretest, 827.63 min MVPA at posttest) than *the z-score* ($\mathrm{z}=\frac{{\mathrm{x}}_{\mathrm{Pre}}-{\mathrm{m}}_{\mathrm{MVPA}}}{{\mathrm{SD}}_{\mathrm{MVPA}}}$ = − 0.18 at pretest, z= $\frac{{\mathrm{x}}_{\mathrm{Post}}-{\mathrm{m}}_{\mathrm{MVPA}}}{{\mathrm{SD}}_{\mathrm{MVPA}}}$ = 0.90 at posttest). Second, a certain increase of PA (e.g. 30 min/week) does not have the same impact on health outcomes as the same decrease in SB (e.g. 30 min/week), regardless of whether standardization is used or not [56].

Descriptive statistics are provided for the total sample and for each of the 8 experimental groups. We checked whether there were significant differences in the sociodemographic variables between participants who chose PA or SB. We also checked whether there were significant differences in the sociodemographic variables and the baseline levels of PA or SB between those who completed the study and those who dropped out (before week 5 or at post-test) using t-tests (continuous variables) or chi-square tests (nominal variables).

Linear mixed effect models with random intercepts for the participant [57] were fitted to the observed data using the *lmer* function from the ‘lme4-package’ [58] in R (version 3.2.5) [59] to investigate the effect of three BCTs (i.e. action planning, coping planning and self-monitoring) and their combinations on either PA or SB [57]. The *p*-values were obtained using the ‘lmerTest-package’. Linear mixed effect models are available case methods, that use all available information to estimate means and covariances and can therefore accommodate missing data points in repeated measures [60] provided that data are assumed to be missing at random. We initially planned to impute missing outcome data, in later meetings with statistical experts however, we chose not to do so for the following reasons. From a statistical point of view, mixed models without ad hoc imputation provide equal or more power than mixed models with ad hoc imputation [61]. Furthermore, using imputation is not advised when the proportions of data that are missing are too large (e.g. more than 40%) [62]. From a theoretical perspective, behaviour change techniques will only be effective if participants are motivated with the intervention and consequently, actually receive these techniques. In real life settings, non-motivated participants will also drop-out of the intervention. Imputing this data could lead to an underestimation of the efficacy of the behaviour change techniques.

There were separate analyses for MVPA and SB. In a first step, for each of the three BCTs (i.e. action planning, coping planning, self-monitoring) the interaction between time (T0 = 0, T1 = 1) and BCT (absent = 0, present = 1) was analysed. This interaction effect examines the efficacy of each BCT, independent from the other BCTs, and provides evidence for *the overall efficacy of each of the three techniques* [63]. In a second step, a model for MVPA (and SB, respectively) was fitted with all possible interactions: time (T0 = 0, T1 = 1) x action planning (absent = 0, present = 1) x coping planning (absent = 0, present = 1) x self-monitoring (absent = 0, present = 1). Based on this model, *pairwise comparisons* between different combinations of the BCTs were analysed. By doing so, we examined to what extent adding a BCT increased efficacy (e.g. the effect of action planning alone can result in a different outcome than for example action planning combined with coping planning). The *pairwise comparisons* were calculated using the *linear hypothesis* function of the ‘car-package’ in R [64]. These ‘follow-up’ or ‘post-hoc’ tests compare the different combinations of the techniques (represented by the 8 experimental groups). The codes for the linear hypotheses, can be found in Additional file 5. Effect sizes (with corresponding 95% confidence intervals) are expressed as the difference in change from pre-test to post-test between combinations.

In order to assess the effects of all possible combinations we had to perform multiple statistical tests. The multiple testing problem can be avoided by adjusting the cut-off score α using the Bonferroni correction to $\frac{\upalpha}{n}$ with *n* being the number of tests performed [65, 66]. However, we chose not to make such adjustments for multiple comparisons because this 1) will lead to fewer errors of interpretation and 2) will not lead to missing possibly important findings [65, 66]. As such, *p*-values below .05 were considered statistically significant.

## Results

### Flow of the study

**Fig. 2:**
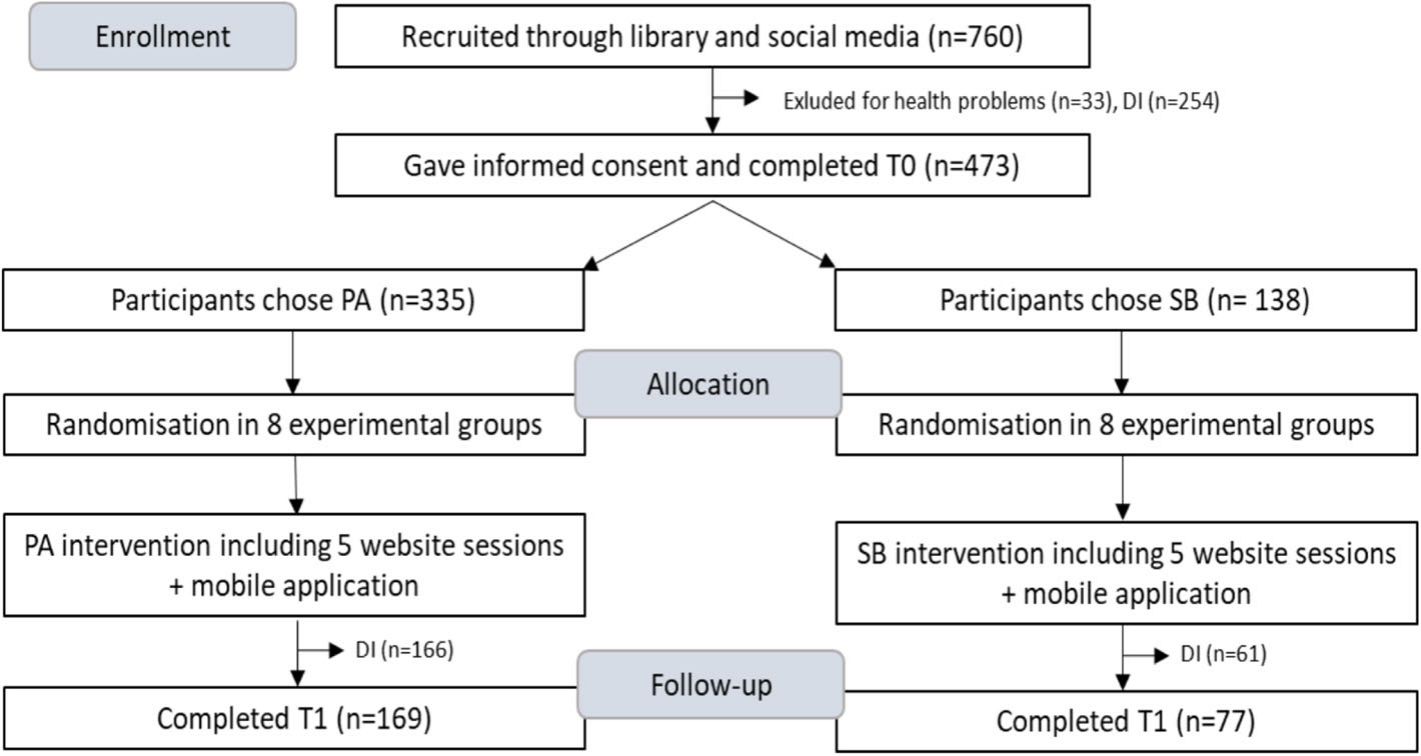
Flow of the participants throughout the study. DI = discontinued, T0 = pre-test measurement, T1 = post-test measurement

The flow of participants throughout the study can be found in Fig. 2. Participants were recruited at the library of Ghent (*n* = 710) and through social media (*n* = 50). In total, 473 individuals agreed to participate, gave their informed consent and completed pre-test measurement. Of these participants, 335 chose to increase their PA and 138 chose to decrease their SB. There were no significant differences in sociodemographic variables between participants who chose PA or SB. Of those who chose PA, 166 dropped out (49,5%). Of those who chose SB, 61 dropped out (44,2%). In our study, non-usage attrition (i.e. not completing the 5 website sessions) automatically equalled drop-out attrition (i.e. not completing post-test measurement T1) since it was impossible for participants to skip certain parts of the intervention because of the linear design of the study (e.g. it was not possible to start for example with session 4 on the website if session 3 was not completed) [34, 37].

The results related to PA and SB are reported separately. The first section describes the effects on PA, the second section describes the effects on SB.

### Physical activity

#### Participant characteristics

Characteristics of participants who chose PA are provided in Table 3. No significant difference in sociodemographic variables or MVPA at baseline was detected between those who completed the study and those who dropped out.

**Table 3:** Characteristics of participants of the PA intervention

**Physical activity**	Total Sample (*n* = 335)	Comb A + C + S (=group 1) *(n = 38)*	Comb A + C (=group 2) *(n = 44)*	Comb A + S (=group 3) *(n = 39)*	Comb C + S (=group 4) *(n = 41)*	A alone (=group 5) *(n = 40)*	C alone (=group 6) *(n = 41)*	S alone (=group 7) *(n = 46)*	No techniques (=group 8) *(n = 46)*
**Age (mean ± SD years)**	35.76 ± 16.48	37.74 ± 16.32	35.66 ± 15.83	33.59 ± 15.73	37.44 ± 16.75	36.78 ± 16.56	36.20 ± 17.83	35.76 ± 16.58	33.33 ± 16.93
**Sex (% male)**	28.1	36.8	36.4	20.5	34.1	25.0	22.0	30.4	19.6
**Level of education (% high = university/college)**	63.3	68.4	61.4	56.4	63.4	64.0	75.6	58.7	58.7
**BMI (mean ± SD kg/m**^**2**^**)**	23.71 ± 3.90	24.60 ± 3.68	24.88 ± 4.11	24.25 ± 4.14	24.49 ± 4.02	22.35 ± 4.02	23.07 ± 3.47	23.70 ± 3.94	22.49 ± 3.16
**MVPA (mean [CI] in min/week)** - **Pretest**	495.04 [420.10; 569.99]	387.42 [239.60; 535.25]	645.11 [433.12; 857.10]	598.51 [398.05; 798.97]	492.85 [289.91; 695.80]	587.63 [374.11; 801.14]	404.98 [210.58; 599.37]	539.97 [355.71; 724.20]	461.22 [300.73; 621.71]
- **Posttest**	603.8 [522.68; 684.89]	827.63 [537.88; 1117.38]	762.06 [445.38; 1078.74]	674.33 [273.25; 1075.41]	647.62 [365.91; 929.83]	370.90 [202.84; 538.98]	476.25 [222.63; 729.87]	663.65 [393.03; 934.27]	491.73 [303.42; 680.04]

SD standard deviation, CI 95% confidence interval, A action planning, C coping planning, S self-monitoring

#### Effects on physical activity as a function of behaviour change technique

In the first step, we investigated the *overall efficacy of each of the three techniques*. The relevant descriptive information of the interaction effects between time and each of the three BCTs are provided for MVPA. The means and 95% confidence intervals for each interaction effect are displayed in Table 4. Analyses indicated that the interaction effect between time and action planning on MVPA was not significant (*t (221.88) = − 0.131*, *p = 0.895*) (Table 4). The increase in MVPA from pre-test to post-test was significantly higher when receiving coping planning versus when not receiving coping planning (*t (218.424) = 2.302*, *p = 0.022*), and when receiving self-monitoring versus not receiving self-monitoring (*t (216.63) = 2.735, p = 0.007)*, respectively (Table 4).

**Table 4:** The interaction effects between time and each of the three BCTs on MVPA. Means and 95% confidence intervals are displayed

**MVPA (min/week)**		Groups who did not receive the technique	Groups who received the technique	t-value (df)	p-value	Effect size
Action planning	Pre-test	476.24 [384.13; 568.34]	558.72 [459.27; 658.17]	−0.131 (221.88)	0.895	−10.73 [− 170.68; 149.23]
	Post-test	574.11 [448.97; 699.26]	643.21 [494.29.93; 792.14]			
Coping planning	Pre-test	543.28 [449.25; 637.31]	487.30 [389.68; 584.93]	2.302 (218.424)	0.022 ^**a**^	184.49 [27.41; 341.56]
	Post-test	542.86 [416.61; 669.12]	671.93 [527.52; 816.36]			
Self-monitoring	Pre-test	524.62 [426.49; 622.75]	506.76 [413.38; 600.15]	2.735 (216.63)	0.007 ^**b**^	217.45 [61.63; 373.34]
	Post-test	510.88 [394.83; 626.94]	698.15 [548.44; 847.87]			

^a^ p < 0.05, ^b^ *p* < 0.01 = significant differences in MVPA from pre-test to post-test between groups who did not receive the technique and groups who received the technique. 95% confidence intervals are shown in square brackets. Df = degrees of freedom. Effect size = absolute effect size in min/week

**Fig. 3:**
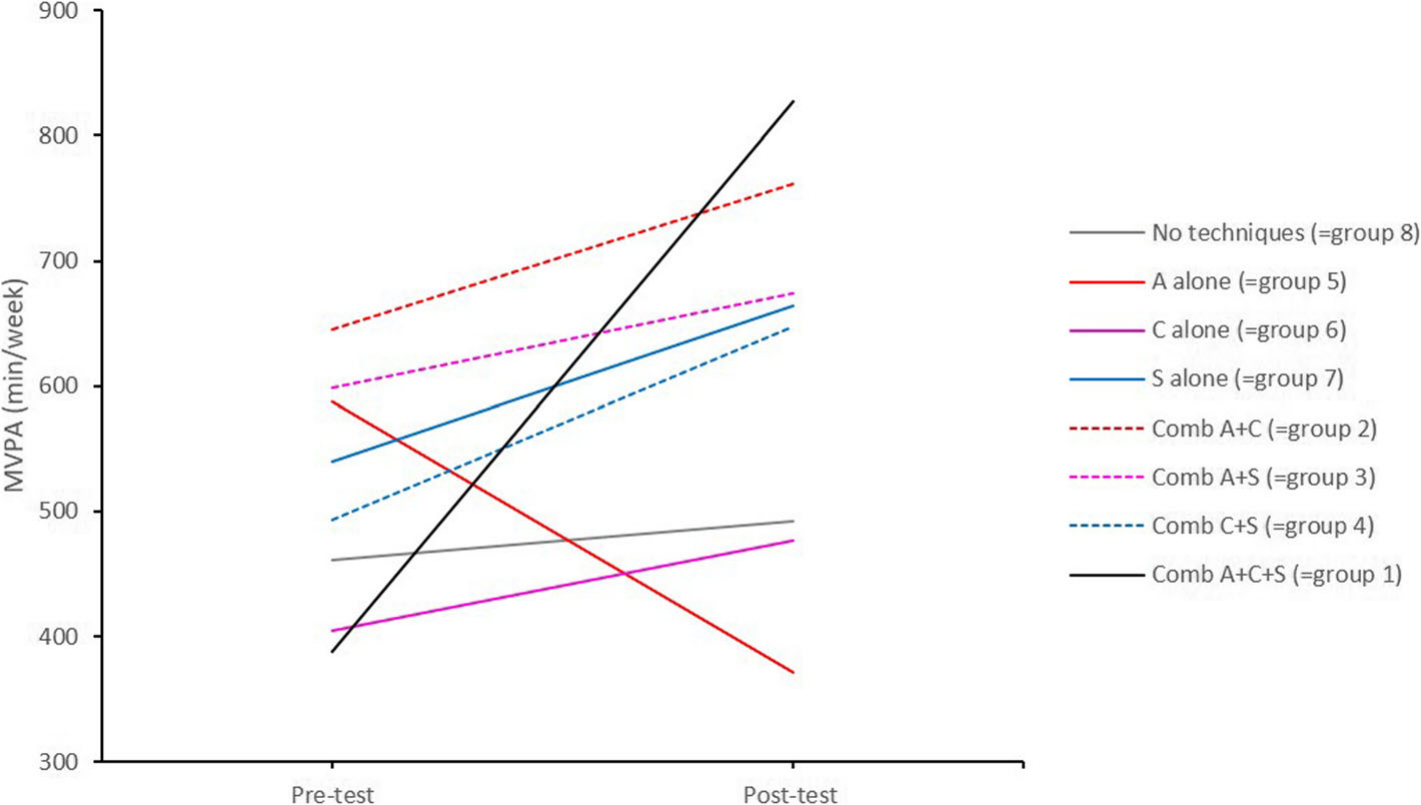
Average MVPA at pre- and post-test for each of the eight experimental groups. Comb = combination, A = action planning, C = coping planning, S = self-monitoring

**Fig. 4:**
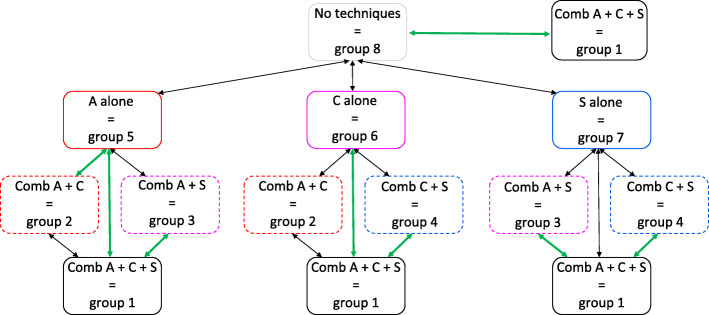
Comparison between the different combinations of the techniques (represented by each of the 8 experimental groups) based on the average difference in MVPA at pre- and post-test. Black arrow = no significant difference between the experimental groups in MVPA from pre- to post-test, *p* > 0.05. Green arrow = significant difference between the experimental groups in MVPA from pre- to post-test, *p* < 0.05

In a second step, an additive approach to identify the effective BCTs or their combinations was used. We investigated to what extent adding a BCT, increased efficacy by using *pairwise comparisons* between each of the eight experimental groups. Figure 3 shows the plot of the average outcomes of MVPA at pre- and post-test for each combination of none, one, two or all techniques (represented by the 8 experimental groups). Table 3 provides the means and 95% confidence intervals for each experimental group at each of the two time points. Figure 4 provides the comparison between the different combinations of the techniques based on the average difference in MVPA from pre- to post-test.

The *pairwise comparisons* are described following the structure of Fig. 4, starting at the top of the figure and then from the left to the right side. 1) The increase in MVPA was significantly larger for the combination A + C + S (=group 1) (*x*^2^ *= 8.879, p = 0.0028)* than for the group who received none of the three BCTs (=group 8). 2) No significant differences in MPVA from pre- to post-test were identified between the group who received none of the three BCTs (=group 8) and the groups who received A alone (=group 5), C alone (=group 6), or S alone (=group 7). 3) The increase in MVPA was significantly larger for the combination A + C (=group 2) (*x*^2^ *= 5.95, p = 0.014*) and the combination A + C + S (=group 1) (*x*^2^ *=17.72, p < 0.001*) than for the group who received A alone (=group 5). Furthermore, the increase in MVPA was significantly larger for the combination A + C + S (=group 1) (*x*^2^ *= 4.552, p = 0.033*) than for the combination A + S (=group 3). 4) The increase in MVPA was significantly larger for the combination A + C + S (=group 1) (*x*^2^ *= 4.435, p = 0.035*) than for the group who received C alone (=group 6). Moreover, the increase in MVPA was significantly larger for the combination A + C + S (=group 1) (*x*^2^ *= 4.094, p = 0.043*) than for the combination C + S (=group 4). 5) No significant differences in MVPA from pre- to post-test were identified between the group who received S alone (=group 7) and the groups receiving the combination A + S (=group 3), the combination C + S (=group 4) or the combination A + C + S (=group 1). 6) Additional analyses (not shown in Fig. 4) showed a significant decrease in MVPA for action planning alone (=group 5) compared with coping planning alone (=group 6) (*x*^2^ *= 4.389*, *p* = *0.031*) and self-monitoring alone (=group 7) (x^2^ = 8.858, *p* = 003), respectively. The outcomes and effect sizes for all pairwise comparisons are provided in Additional file 5.

### Sedentary behaviour

#### Participants characteristics

Characteristics of the participants who chose SB are provided in Table 5. No significant difference in sociodemographic variables or SB at baseline was detected between those who completed the study and those who dropped out.

**Table 5:** Characteristics of participants of the SB intervention

**Sedentary Behaviour**	Total Sample (*n* = 138)	Comb A + C + S (=group 1) *(n = 21)*	Comb A + C (=group 2) *(n = 16)*	Comb A + S (=group 3) *(n = 17)*	Comb C + S (=group 4) *(n = 15)*	A alone (=group 5) *(n = 21)*	C alone (=group 6) *(n = 18)*	S alone (=group 7) *(n = 15)*	No techniques (=group 8) *(n = 15)*
**Age (mean ± SD years)**	37.80 ± 15.98	37.71 ± 14.47	33.25 ± 15.85	38.53 ± 15.63	44.13 ± 18.13	37.33 ± 15.94	38.16 ± 15.87	38.67 ± 16.66	34.93 ± 17.27
**Sex (% male)**	37.7	52.4	31.2	29.4	0.33	47.6	27.8	26.7	46.7
**Level of education (% high = university/college)**	73.9	85.7	62.5	76.5	73.3	85.7	88.9	53.3	53.3
**BMI (mean ± SD kg/m**^**2**^**)**	23.02 ± 3.25	23.12 ± 3.04	21.01 ± 2.81^a^	24.25 ± 3.24	24.74 ± 2.87^a^	23.13 ± 3.76	22.97 ± 3.14	22.48 ± 3.27	22.36 ± 2.89
**SB (mean [CI] in hrs/day)** **- Pretest**	12.02 [11.36; 12.69]	12.51 [11.04; 13.99]	12.91 [11.17; 14.66]	12.52 [10.61; 14.43]	11.98 [10.05; 13.91]	11.51 [10.00; 13.03]	11.43 [10.39; 12.47]	11.83 [10.22; 13.45]	12.05 [10.16; 13.95]
**- Posttest**	10.23 [9.55; 10.91]	9.88 [7.54; 12.22]	10.96 [8.90; 13.01]	9.06 [6.40; 11.72]	9.65 [8.01; 11.31]	10.72 [8.41; 13.03]	11.06 [9.07; 13.06]	10.10 [8.05; 12.15]	10.51 [7.59; 13.44]

SD standard deviation, CI 95% confidence interval, A action planning, C coping planning, S self-monitoring

#### Effects on sedentary behaviour as a function of behaviour change technique

In the first step, we investigated *the overall efficacy of each of the three techniques*. The relevant descriptive information of the interaction effects between time and each of the three BCTs are provided for SB. The means and 95% confidence intervals for each BCT are displayed in Table 6. Analyses indicated that the interaction effect between time and action planning (*t (103.94) = − 0.952, p = 0.343*) and the interaction effect between time and coping planning (*t (104.05) = − 0.084, p = 0.933*) on SB were not significant (Table 6). The decrease in SB from pre-test to post-test was significantly higher when receiving self-monitoring versus when not receiving self-monitoring (*t (100.66) = − 2.573, p = 0.012*) (Table 6).

**Table 6:** The interaction effects between time and each of the three BCTs on SB. Means and 95% confidence intervals are displayed

**SB (hours/day)**		Groups who did not receive the technique	Groups who received the technique	t-value (df)	p-value	Effect size
Action planning	Pre-test	11.80 [11.01; 12.60]	12.32 [11.50; 13.14]	−0.952 (103.94)	0.343	−0.64 [−1.95; 0.67]
	Post-test	10.30 [9.25; 11.36]	10.17 [9.01; 11.35]			
Coping planning	Pre-test	11.95 [11.10; 12.81]	12.21 [11.55; 12.98]	−0.084 (104.05)	0.933	−0.06 [−1.37; 1.26]
	Post-test	10.14 [8.93; 11.35]	10.33 [9.29; 11.37]			
Self-monitoring	Pre-test	11.93 [11.15; 12.70]	12.25 [11.40; 13.09]	−2.573 (100.66)	0.012 ^a^	−1.66 [−2.93; −0.40]
	Post-test	10.80 [9.67; 11.94]	9.70 [8.62; 10.79]			

^a^ p < 0.05 = significant differences in SB from pre- to post-test between groups who did not receive the technique and groups who received the technique. Ninety five percent confidence intervals are shown in square brackets. Df = degrees of freedom. Effect size = absolute effect size in hours/day

**Fig. 5:**
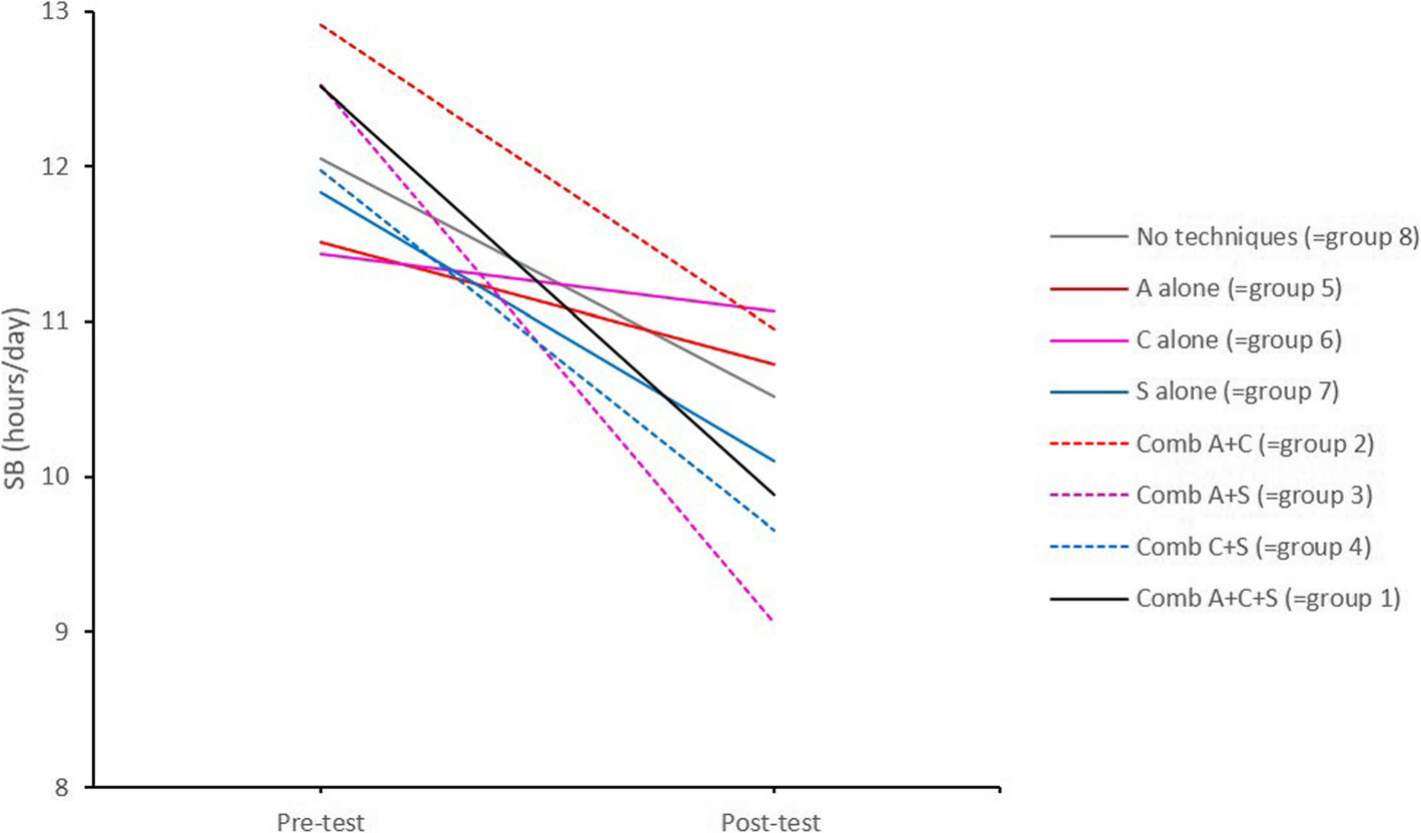
Average SB at pre- and post-test for each of the eight experimental groups. Comb = combination, A = action planning, C = coping planning, S = self-monitoring

**Fig. 6:**
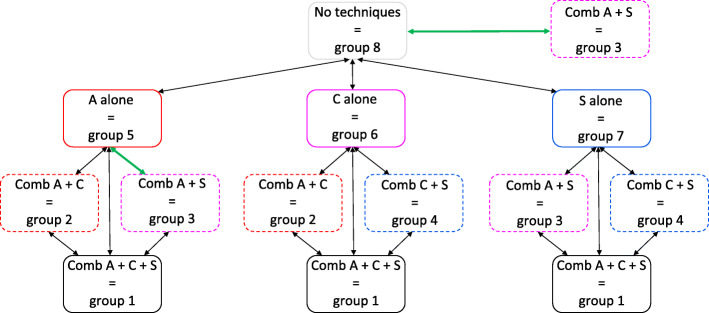
Comparison between the different combinations of the techniques (represented by each of the 8 experimental groups) based on the average difference in SB at pre- and post-test. Black arrow = no significant difference between the experimental groups in SB from pre-test to post-test measurement, p > 0.05. Green arrow = significant difference between the experimental groups in SB from pre-test to post-test measurement, p < 0.05

In a second step, an additive approach to identify the effective BCTs or their combinations was used. We investigated to what extent adding a BCT, increased efficacy by using *pairwise comparisons* between each of the eight experimental groups. Figure 5 shows the plot of the average outcomes of SB at pre- and post-test for each combination of none, one, two or all techniques (represented by the 8 experimental groups). Table 5 provides the means and 95% confidence intervals for each experimental group at each of the two time points. Figure 6 provides the comparison between the different combinations of the techniques based on the average difference in SB from pre- and post-test.

The *pairwise comparisons* are described following the structure of Fig. 6, starting at the top of the figure and then from the left to the right side. 1) The decrease in SB was significantly larger for the combination A + S (=group 3) (*x*^2^ *= 3.917, p = 0.048)* than for the group who received no techniques (=group 8). 2) No significant differences in SB from pre- to post-test were identified between the group who received no techniques and the groups who received A alone (=group 5), C alone (=group 6) or S alone (=group 7). 3) The decrease in SB was also significantly larger for the combination A + S (=group 3) (*x*^2^ *= 5.761, p = 0.016)* than for the group who received A alone (=group 5). 4) No significant differences in SB from pre- to post-test were identified for C and its combinations. 5) No significant differences in SB from pre- to post-test were identified for S and its combinations. The outcomes and effect sizes for all pairwise comparisons are provided in Additional file 5.

## Discussion

Behaviour change interventions are complex interventions, including a range of techniques [67]. Meta-analyses have shown that these interventions work, and also suggest that certain techniques may be more effective than others (e.g. self- monitoring) [31, 68]. However, the conclusion that certain techniques work better than others is preliminary, and await experimental corroboration [32]. Using a 2x2x2 factorial study [63], the presence of *three BCTs that target post-intentional processes* (i.e. action planning, coping planning, self-monitoring) was experimentally manipulated, and their effects upon PA and SB were investigated. These experimental manipulations were implemented on top of a basic intervention, which all participants received. In a first step, we examined *the overall efficacy of each of three BCTs*. In a second step, we used an additive approach to identify the effective BCTs or their combinations. We investigated to what extent *adding BCTs increased efficacy*.

The pattern of results indicates that an intervention including self-monitoring is overall more effective than an intervention without self-monitoring for both MVPA and SB. This is in line with research showing that self-monitoring is a critical technique for behaviour change [31]. It may be sufficient to change behaviour, as for example in reducing SB [68, 69]. For other behaviours, other techniques may also be needed. For example, we may expect that increasing MVPA requires planning. In this study, we found that an intervention including coping planning was overall more effective in changing MVPA. For action planning, no overall effect was found. Previous research has also found no or only moderate effects for action planning, in particular when accounting for the effects of past behaviour [70, 71]. A possible explanation is that individuals may not need to explicitly form plans, but that the intention to be physically active is sufficient to initiate previously developed behavioural schema. The efficacy of action planning could then wane as the intervention proceeds. In line with this possibility, action planning has been found to be especially efficacious for intenders (individuals that intend to, but not yet execute the behaviour) as opposed to actors (individuals that already execute the behaviour) [72, 73]. The inclusion of coping planning in an intervention did have a significant effect. This is remarkable because in a previous study, the use of coping plans has been shown to frustrate users, and many users provided coping plans of low quality [46]. At least two explanations may account for the overall effect of coping planning. First, in our intervention participants were approached as their own expert and received more guidance than in previous versions of MyPlan. Coping plans were constructed by filling out questions and several examples were provided. It thus may well be that coping planning turned out to be effective, because of the proper guidance in coping planning. Second, coping planning may have prompted action planning. Indeed, in contrast to pharmacological interventions, individuals may spontaneously make use of particular BCTs [74, 75], even when the BCTs are not delivered in the intervention.

Of further importance to this study was a detailed analysis of which BCTs or their combination were more effective. Such approach is much needed and warranted to provide guidance in developing efficient interventions [33]. Before providing the results in more detail, a cautionary note should be kept in mind. Despite our efforts to have a large number of participants in each experimental group, statistical power may be low to detect significant differences. This is particularly the case for sedentary behaviour: the number of participants in each group was in any group not larger than 21. Hence, in interpreting the results below one should not confuse ‘no evidence for an effect’ with ‘evidence for no effect’.

For **MVPA**, results showed that the combination of the three BCTs (action planning, coping planning and self-monitoring) were required to result in an effective intervention (i.e. larger difference from pre-test to post-test in MVPA compared to the group without these three BCTs). Increasing MVPA may thus require more than only self-monitoring. Interestingly, action planning is more effective when combined with coping planning. This is in line with previous research [76, 77]. Coping planning, however, was not more effective when combined with action planning. As mentioned above, it might be that individuals spontaneously start to plan actions when invited to develop a coping plan. A remarkable finding was that action planning was less effective than the other two techniques. Action planning even decreased PA (on average 216.73 min/week **less** PA after vs. before the intervention), whereas self-monitoring and coping planning each increased PA (respectively on average 123.68 and 71.27 min/week **more** PA after vs. before the intervention). We do not yet have a full explanation for this finding. A possible one may be found in Maher & Conroy [78], who revealed that action planning is a useful BCT for increasing PA in individuals with weak habits, but may decrease PA in individuals with strong habits. Most people in our sample were already quite physically active at the start of the study, and might thus have had strong habits with respect to PA. Explicit efforts to manipulate these habits may have backfired. These results, together with those of previous studies suggest that action planning, at least when implemented alone, should be preferably used in people with weak PA habits [78] and rather, as already mentioned above, in intenders as opposed to actors [72, 73].

For **SB**, the combination of action planning and self-monitoring was sufficient for an effective intervention. It may not be surprising that coping planning is of less importance for SB, as standing up does not take much time and is not difficult to achieve. Barriers that do present itself, may be difficult to overcome (e.g. cannot stand up during meeting). Previous studies have already shown that self-monitoring is important to decrease SB [68]. In the present study we found no evidence that self-monitoring alone is efficacious. However, it should be noted that the sample size in the present study was small and that this may have obscured effects. With respect to action planning, De Cocker et al. [79] found that their ‘Start to Stand’ intervention for office workers was only efficacious for those participants completing action plans. Here we showed that including both self-monitoring and action planning might be most beneficial.

### Strengths and limitations

To the best of our knowledge, this is the first study that used an experimental 2x2x2 design to isolate the effects of three post-intentional BCTs (i.e. action planning, coping planning and self-monitoring) and their combinations to promote PA and reduce SB. Until now, other studies investigated their efficacy mostly within multiple BCT interventions or in a non-experimental way. Moreover, the BCTs were tested in a PA intervention as well as a SB intervention, which gave insight into the working mechanisms of both behaviours.

This study also has a number of limitations. First, PA and SB were measured by self-report questionnaires, which may lead to response and recall biases [80]. Future research may consider the use of devices to measure PA and SB [81, 82]. Second, as stated in the protocol paper [37] our initial power calculations were based on the analyses of PA and SB together. After consultation with statistical experts, PA and SB were analysed separately. Unfortunately, the SB intervention had less than half of the participants of the PA intervention. As such, the sample size in the SB intervention was small, probably resulting in a lack of power to detect some differences. Third, there was a high drop-out rate (49.5% in the PA intervention and 44.2% in the SB intervention), despite efforts to accommodate end-users [45, 46]. A high drop-out is often seen in e- and m-health research: Stopping the intervention is only one click away [34, 83]. Using only a subset of BCTs that have shown to be effective might decrease attrition. Therefore, it is important to experimentally identify which BCTs are effective as to reduce attrition in future interventions. Also, e- and m-health interventions are often “one-size-fits all” interventions. A more personalised intervention may increase user engagement and reduce attrition rates, and may occur at two levels. Regarding the first level: individuals might differ in terms of their motivational stages (pre-intender, intender, actor) [21], and these may change over time during the intervention. In the future, interventions should be developed in which the delivery of BCTs are dynamically tailored to these changing demands. For example, action planning may be effective in the beginning of the intervention, but not later on; once physical activity has become a habit, action planning might become ineffective, or even counterproductive [78, 84]. Regarding the second level: The delivery of the BCT itself (e.g. the support to make qualitative action and coping plans) is often generic, and not contextualized and personalised to the individual. As such, there is a need to provide a more contextualized and personalised support in this process [85]. Fourth, this study only experimentally manipulated action planning, coping planning and self-monitoring. Other techniques (e.g. providing information about the consequences of behaviour, feedback on performance, social support, reviewing behaviour goals) were also present, but not experimentally manipulated. As such, one should interpret the efficacy of our experimentally manipulated BCTs against the background of these BCTs.

## Supplementary information

**Additional file 1.** Completed CONSORT 2010 checklist.

**Additional file 2.** Completed TIDieR checklist.

**Additional file 3.** Screenshots of the website.

**Additional file 4.** Screenshots of the mobile application.

**Additional file 5.** The codes of the linear hypotheses and the outcomes with effect sizes for all pairwise comparisons for MVPA and SB.

## Data Availability

Data supporting the results reported in this article are stored at the University of Ghent, Belgium. The datasets used during the current study are available from the corresponding author on reasonable request.

## Abbreviations

A: Action planning
BCT: Behaviour change technique
C: Coping planning
IPAQ: International Physical Activity Questionnaire
MVPA: Moderate to vigorous physical activity
PA: Physical activity
PAR-Q: Physical Activity Readiness Questionnaire
SB: Sedentary behaviour
S: Self-monitoring
SIT-Q-7d: Last 7-days Sedentary Behaviour Questionnaire
T0: Pre-test
T1: Post-test
