# Supplementary material for: Which behaviour change techniques are effective to promote physical activity and reduce sedentary behaviour in adults: a factorial randomized trial of an e- and m-health intervention
Source: Int J Behav Nutr Phys Act. 2020 Oct 7;17:127. doi: 10.1186/s12966-020-01001-x (PMC7539442; doi:10.1186/s12966-020-01001-x)
Supplement: Supplementary file 5 — Additional file 5. The codes of the linear hypotheses and the outcomes with effect sizes for all pairwise comparisons for MVPA and SB. [file 12966_2020_1001_MOESM5_ESM.pdf]

	Time 0 (No techniques= group 8)	Time 1 (No techniques= group 8)	**SLOPE No techniques** (= group 8)	Time 0 (A alone= group 5)	Time 1 (A alone= group 5)	**SLOPE A alone** (= group 5)	Time 0 (C alone= group 6)	Time 1 (C alone= group 6)	**SLOPE C alone** (= group 6)	Time 0 (S alone= group 7)	Time 1 (S alone= group 7)	**SLOPE S alone** (= group 7)
1. Intercept	1	1	0	1	1	0	1	1	0	1	1	0
2. Time	0	1	1	0	1	1	0	1	1	0	1	1
3. ActionPlanning	0	0	0	1	1	0	0	0	0	0	0	0
4. CopingPlanning	0	0	0	0	0	0	1	1	0	0	0	0
5. SelfMonitoring	0	0	0	0	0	0	0	0	0	1	1	0
6. Time:ActionPlanning	0	0	0	0	1	1	0	0	0	0	0	0
7. Time:CopingPlanning	0	0	0	0	0	0	0	1	1	0	0	0
8. ActionPlanning:CopingPlanning	0	0	0	0	0	0	0	0	0	0	0	0
9. Time:Selfmonitoring	0	0	0	0	0	0	0	0	0	0	1	1
10. ActionPlanning:SelfMonitoring	0	0	0	0	0	0	0	0	0	0	0	0
11. Copingplanning:SelfMonitoring	0	0	0	0	0	0	0	0	0	0	0	0
12. Time:ActionPlanning:CopingPlanning	0	0	0	0	0	0	0	0	0	0	0	0
13. Time:ActionPlanning:SelfMonitoring	0	0	0	0	0	0	0	0	0	0	0	0
14. Time:CopingPlanning:Selfmonitoring	0	0	0	0	0	0	0	0	0	0	0	0
15. AcionPlanning:CopingPlanning:SelfMonitoring	0	0	0	0	0	0	0	0	0	0	0	0
16. Time:ActionPlanning:CopingPlanning:SelfMonitoring	0	0	0	0	0	0	0	0	0	0	0	0
**MVPA**	/	/	/	/	/	/	/	/	/	/	/	/
**SB**	/	/	/	/	/	/	/	/	/	/	/	/

Additional file 5. The codes of the linear hypotheses and the outcomes with effect sizes for all pairwise comparisons for MVPA and SB. A "SLOPE" is calculated by the difference between Time 1 and Time 0. Time 0= pretest, Time 1= posttest. A= action planning, C= coping planning, S= self-monitoring, Comb= combination. *P<0.05, **P<0,01= significant differences in MVPA/SB from pretest to posttest between the different combinations of techniques (represented by each of the 8 groups). ES [CI]= effect size with 95% confidence interval in min/week for MVPA and hours/day for SB.

	Time 0 (Comb A + C= group 2)	Time 1 (Comb A + C= group 2)	**SLOPE Comb A+C** (= group 2)	Time 0 (Comb A + S= group 3)	Time 1 (Comb A + S= group 3)	**SLOPE Comb A+S** (= group 3)	Time 0 (Comb C + S= group 4)	Time 1 (Comb C + S= group 4)	**SLOPE Comb C+S** (= group 4)	Time 0 (Comb A+C+S= group 1)	Time 1 (Comb A+C+S= group 1)	**SLOPE Comb A+C+S** (= group 1)
1. Intercept	1	1	0	1	1	0	1	1	0	1	1	0
2. Time	0	1	1	0	1	1	0	1	1	0	1	1
3. ActionPlanning	1	1	0	1	1	0	0	0	0	1	1	0
4. CopingPlanning	1	1	0	0	0	0	1	1	0	1	1	0
5. SelfMonitoring	0	0	0	1	1	0	1	1	0	1	1	0
6. Time:ActionPlanning	0	1	1	0	1	1	0	0	0	0	1	1
7. Time:CopingPlanning	0	1	1	0	0	0	0	1	1	0	1	1
8. ActionPlanning:CopingPlanning	1	1	0	0	0	0	0	0	0	1	1	0
9. Time:Selfmonitoring	0	0	0	0	1	1	0	1	1	0	1	1
10. ActionPlanning:SelfMonitoring	0	0	0	1	1	0	0	0	0	1	1	0
11. Copingplanning:SelfMonitoring	0	0	0	0	0	0	1	1	0	1	1	0
12. Time:ActionPlanning:CopingPlanning	0	1	1	0	0	0	0	0	0	0	1	1
13. Time:ActionPlanning:SelfMonitoring	0	0	0	0	1	1	0	0	0	0	1	1
14. Time:CopingPlanning:Selfmonitoring	0	0	0	0	0	0	0	1	1	0	1	1
15. AcionPlanning:CopingPlanning:SelfMonitoring	0	0	0	0	0	0	0	0	0	1	1	0
16. Time:ActionPlanning:CopingPlanning:SelfMonitoring	0	0	0	0	0	0	0	0	0	0	1	1
**MVPA**	/	/	/	/	/	/	/	/	/	/	/	/
**SB**	/	/	/	/	/	/	/	/	/	/	/	/

	Difference slope no techniques and A alone (group 8 vs 5)	Difference slope no techniuqes and C alone (group 8 vs 6)	Difference slope no techniques and S alone (group 8 vs 7)
1. Intercept	0	0	0
2. Time	0	0	0
3. ActionPlanning	0	0	0
4. CopingPlanning	0	0	0
5. SelfMonitoring	0	0	0
6. Time:ActionPlanning	1	0	0
7. Time:CopingPlanning	0	1	0
8. ActionPlanning:CopingPlanning	0	0	0
9. Time:Selfmonitoring	0	0	1
10. ActionPlanning:SelfMonitoring	0	0	0
11. Copingplanning:SelfMonitoring	0	0	0
12. Time:ActionPlanning:CopingPlanning	0	0	0
13. Time:ActionPlanning:SelfMonitoring	0	0	0
14. Time:CopingPlanning:Selfmonitoring	0	0	0
15. AcionPlanning:CopingPlanning:SelfMonitoring	0	0	0
16. Time:ActionPlanning:CopingPlanning:SelfMonitoring	0	0	0
**MVPA**	P= 0,151<br>x^2= 2,067<br>ES [CI]= -216,24 [-491,96; 59,49]	P= 0,443<br>x^2= 0,589<br>ES [CI]= 101,94 [-151,00; 384,88]	P= 0,109<br>x^2= 2,576<br>ES [CI]= 202,53 [-75,53; 480,59]
**SB**	P= 0,829<br>x^2= 0,047<br>ES [CI]= 0,06 [-2,05; 2,18]	P= 0,608<br>x^2= 0,263<br>ES [CI]= 0,90 [-1,86; 3,67]	P= 0,560<br>x^2= 0,340<br>ES [CI]= -0,88 [-3,28; 1,51]

	Difference slope no techniques and Comb A+C (group 8 vs 2)	Difference slope no techniques and Comb A+S (group 8 vs 3)	Difference slope no techniques and Comb C+S (group 8 vs 4)
1. Intercept	0	0	0
2. Time	0	0	0
3. ActionPlanning	0	0	0
4. CopingPlanning	0	0	0
5. SelfMonitoring	0	0	0
6. Time:ActionPlanning	1	1	0
7. Time:CopingPlanning	1	0	1
8. ActionPlanning:CopingPlanning	0	0	0
9. Time:Selfmonitoring	0	1	1
10. ActionPlanning:SelfMonitoring	0	0	0
11. Copingplanning:SelfMonitoring	0	0	0
12. Time:ActionPlanning:CopingPlanning	1	0	0
13. Time:ActionPlanning:SelfMonitoring	0	1	0
14. Time:CopingPlanning:Selfmonitoring	0	0	1
15. AcionPlanning:CopingPlanning:SelfMonitoring	0	0	0
16. Time:ActionPlanning:CopingPlanning:SelfMonitoring	0	0	0
**MVPA**	P= 0,242<br>x^2= 1,370<br>ES [CI]= 179,53 [-119,31; 478,36]	P= 0,591<br>x^2= 0,289<br>ES [CI]= 91,61 [-190,64; 373,86]	P= 0,331<br>x^2= 0,945<br>ES [CI]= 141,48 [-97,98; 380,94]
**SB**	P= 0,623<br>x^2= 0,233<br>ES [CI]= -0,64 [-3,38; 2,10]	P= 0,048*<br>x^2= 3,918<br>ES [CI]= -2,44 [-5,70; -0,82]	P= 0,286<br>x^2= 1,139<br>ES [CI]= -1,36 [-4,21; 1,47]

	Difference slope no techniques and Comb A+C+S (group 8 vs 1)	Difference slope A alone and Comb A+C (group 5 vs 2)	Difference slope A alone and Comb A+S (group 5 vs 3)
1. Intercept	0	0	0
2. Time	0	0	0
3. ActionPlanning	0	0	0
4. CopingPlanning	0	0	0
5. SelfMonitoring	0	0	0
6. Time:ActionPlanning	1	0	0
7. Time:CopingPlanning	1	1	0
8. ActionPlanning:CopingPlanning	0	0	0
9. Time:Selfmonitoring	1	0	1
10. ActionPlanning:SelfMonitoring	0	0	0
11. Copingplanning:SelfMonitoring	0	0	0
12. Time:ActionPlanning:CopingPlanning	1	1	0
13. Time:ActionPlanning:SelfMonitoring	1	0	1
14. Time:CopingPlanning:Selfmonitoring	1	0	0
15. AcionPlanning:CopingPlanning:SelfMonitoring	0	0	0
16. Time:ActionPlanning:CopingPlanning:SelfMonitoring	1	0	0
**MVPA**	P= 0,003*<br>x^2= 8,849<br>ES [CI]= 424,80 [125,89; 723,72]	P= 0,014*<br>x^2= 5,950<br>ES [CI]= 388,85 [60,80; 716,90]	P= 0,074<br>x^2= 3,180<br>ES [CI]= 295,25 [-15,43; 605,93]
**SB**	P= 0,243<br>x^2= 1,365<br>ES [CI]= -1,32 [-4,33; 1,70]	P= 0,451<br>x^2= 0,568<br>ES [CI]= -0,77 [-2,48; 0,94]	P= 0,016*<br>x^2= 5,761<br>ES [CI]= -3,03 [-5,22; -0,85]

	Difference slope A alone and Comb A+C+S (group 5 vs 1)	Difference slope C alone and Comb A+C (group 6 vs 2)	Difference slope C alone and Comb C+S (group 6 vs 4)
1. Intercept	0	0	0
2. Time	0	0	0
3. ActionPlanning	0	0	0
4. CopingPlanning	0	0	0
5. SelfMonitoring	0	0	0
6. Time:ActionPlanning	0	1	0
7. Time:CopingPlanning	1	0	0
8. ActionPlanning:CopingPlanning	0	0	0
9. Time:Selfmonitoring	1	0	1
10. ActionPlanning:SelfMonitoring	0	0	0
11. Copingplanning:SelfMonitoring	0	0	0
12. Time:ActionPlanning:CopingPlanning	1	1	0
13. Time:ActionPlanning:SelfMonitoring	1	0	0
14. Time:CopingPlanning:Selfmonitoring	1	0	1
15. AcionPlanning:CopingPlanning:SelfMonitoring	0	0	0
16. Time:ActionPlanning:CopingPlanning:SelfMonitoring	1	0	0
**MVPA**	P= 0,00003**<br>x^2= 17,722<br>ES [CI]= 657,28 [326,60; 987,96]	P= 0,682<br>x^2= 0,168<br>ES [CI]= 67,37 [-265,56; 400,31]	P= 0,871<br>x^2= 0,026<br>ES [CI]= 6,83 [-258,48; 272,14]
**SB**	P= 0,123<br>x^2= 2,381<br>ES [CI]= -1,74 [-3,97; 0,50]	P= 0,311<br>x^2= 1,026<br>ES [CI]= -1,41 [-3,78; 0,96]	P= 0,107<br>x^2= 2,560<br>ES [CI]= -2,05 [-4,57; 0,46]

	Difference slope C alone and Comb A+C+S (group 6 vs 1)	Difference slope S alone and Comb A+S (group 7 vs 3)	Difference slope S alone and Comb C+S (group 7 vs 4)
1. Intercept	0	0	0
2. Time	0	0	0
3. ActionPlanning	0	0	0
4. CopingPlanning	0	0	0
5. SelfMonitoring	0	0	0
6. Time:ActionPlanning	1	1	0
7. Time:CopingPlanning	0	0	1
8. ActionPlanning:CopingPlanning	0	0	0
9. Time:Selfmonitoring	1	0	0
10. ActionPlanning:SelfMonitoring	0	0	0
11. Copingplanning:SelfMonitoring	0	0	0
12. Time:ActionPlanning:CopingPlanning	1	0	0
13. Time:ActionPlanning:SelfMonitoring	1	1	0
14. Time:CopingPlanning:Selfmonitoring	1	0	1
15. AcionPlanning:CopingPlanning:SelfMonitoring	0	0	0
16. Time:ActionPlanning:CopingPlanning:SelfMonitoring	1	0	0
**MVPA**	P= 0,035*<br>x^2=4,435<br>ES [CI]= 358,33 [12,00; 704,65]	P= 0,3907<br>x^2= 0,737<br>ES [CI]= - 147,77 [-466,69; 171,15]	P= 0,550<br>x^2= 0,358<br>ES [CI]= -110 [-378,85; 157,88]
**SB**	P= 0,082<br>x^2= 3,024<br>ES [CI]=-2,23 [-4,92; 0,46]	P= 0,141<br>x^2=2,166<br>ES [CI]= -1,90 [-4,41; 0,60]	P= 0,604<br>x^2= 0,269<br>ES [CI]= -0,69 [-2,72; 1,33]

	Difference slope S alone and Comb A+C+S (group 7 vs 1)	Difference slope Comb A+C and slope Comb A+C+S (group 2 vs 1)	Difference slope Comb A+S and slope Comb A+C+S (group 3 vs 1)	Difference slope Comb C+S and slope Comb A+C+S (group 4 vs 1)
1. Intercept	0	0	0	0
2. Time	0	0	0	0
3. ActionPlanning	0	0	0	0
4. CopingPlanning	0	0	0	0
5. SelfMonitoring	0	0	0	0
6. Time:ActionPlanning	1	0	0	1
7. Time:CopingPlanning	1	0	1	0
8. ActionPlanning:CopingPlanning	0	0	0	0
9. Time:Selfmonitoring	0	1	0	0
10. ActionPlanning:SelfMonitoring	0	0	0	0
11. Copingplanning:SelfMonitoring	0	0	0	0
12. Time:ActionPlanning:CopingPlanning	1	0	1	1
13. Time:ActionPlanning:SelfMonitoring	1	1	0	1
14. Time:CopingPlanning:Selfmonitoring	1	1	1	0
15. AcionPlanning:CopingPlanning:SelfMonitoring	0	0	0	0
16. Time:ActionPlanning:CopingPlanning:SelfMonitoring	1	1	1	1
**MVPA**	P= 0,135<br>x²= 2,240<br>ES [CI]= 272,06 [-61,73; 605,85]	P= 0,103<br>x²= 2,666<br>ES [CI]=290,74 [-72,07; 653,56]	P= 0,033*<br>x²= 4,552<br>ES [CI]= 363,20 [20,25; 706,14]	P= 0,043*<br>x²= 4,094<br>ES [CI]= 310,19 [18,50; 601,89]
**SB**	P= 0,5562<br>x²= 0,3463<br>ES [CI]= -0,74 [-3,21; 1,74]	P= 0,499<br>x²= 0,457<br>ES [CI]= -0,77 [-3,75; 1,93]	P= 0,334<br>x²= 0,934<br>ES [CI]= 0,98 [-2,09; 4,07]	P= 0,976<br>x²= 0,001<br>ES [CI]= -0,15 [-2,95; 2,65]

	Difference slope A alone and C alone (group 5 vs 6)	Difference slope A alone and S alone (group 5 vs 7)	Difference slope C alone and S alone (group 6 vs 7)
1. Intercept	0	0	0
2. Time	0	0	0
3. ActionPlanning	0	0	0
4. CopingPlanning	0	0	0
5. SelfMonitoring	0	0	0
6. Time:ActionPlanning	1	1	0
7. Time:CopingPlanning	-1	0	1
8. ActionPlanning:CopingPlanning	0	0	0
9. Time:Selfmonitoring	0	-1	-1
10. ActionPlanning:SelfMonitoring	0	0	0
11. Copingplanning:SelfMonitoring	0	0	0
12. Time:ActionPlanning:CopingPlanning	0	0	0
13. Time:ActionPlanning:SelfMonitoring	0	0	0
14. Time:CopingPlanning:Selfmonitoring	0	0	0
15. AcionPlanning:CopingPlanning:SelfMonitoring	0	0	0
16. Time:ActionPlanning:CopingPlanning:SelfMonitoring	0	0	0
**MVPA**	P= 0,031*<br>x^2= 4,389<br>ES [CI]= 317,59 [6,39; 628,79]	P=0,003**<br>x^2= 8,858<br>ES [CI]= 418,95 [113,61; 724,28]	P=0,460<br>x^2=0,546<br>ES [CI]= 102,81 [-210,20; 415,82]
**SB**	P=0,732<br>x^2= 0,118<br>ES [CI]= 0,43 [-1,36; 2,22]	P=0,384<br>x^2= 0,759<br>ES [CI]= -1,13 [-2,44; 0,18]	P=0,261<br>x^2= 1,262<br>ES [CI]= -1,48 [-3,51; 0,55]
